# Human pregnane X receptor compromises the function of p53 and promotes malignant transformation

**DOI:** 10.1038/cddiscovery.2016.23

**Published:** 2016-04-18

**Authors:** D Robbins, M Cherian, J Wu, T Chen

**Affiliations:** 1 Department of Chemical Biology and Therapeutics, St. Jude Children’s Research Hospital, Memphis, TN, USA

## Abstract

The pregnane X receptor (PXR) is well established as a nuclear receptor that has a central role in xenobiotic metabolism and disposition. However, emerging evidence suggests that PXR is also a regulator of apoptosis, promoting a malignant phenotype both *in vitro* and *in vivo*. The tumor suppressor p53 can be activated in the presence of DNA damage and induce cell cycle arrest to allow for DNA repair or, ultimately, apoptosis to suppress tumor formation. We previously identified p53 as a novel PXR-associated protein by using a mass spectrometric approach. In the current study, we identified a novel inhibitory effect of PXR on p53, revealing an anti-apoptotic function of PXR in colon carcinogenesis. PXR expression reduced p53 transactivation and the expression of its downstream target genes involved in cell cycle arrest and apoptosis by decreasing p53 recruitment to the promoter regions of these genes. Consistent with the inhibitory effect of PXR on p53, elevated PXR levels decreased doxorubicin- or nutlin-3a-mediated toxicity and promoted malignant transformation in colon cancer cells. Our findings show for the first time that PXR expression modulates p53 target gene promoter binding and contributes to the downregulation of p53 function in human colon cancer cells. These results define the functional significance of PXR expression in modulating p53-mediated mechanisms of tumor suppression.

## Introduction

Physiologic homeostasis is partly sustained through the regulation of metabolism and export of xenobiotics that enter the body. The nuclear xenobiotic pregnane X receptor (PXR) is well established as a transcriptional regulator of phase I and phase II drug-metabolizing enzymes and drug transporters and is highly expressed in liver and small intestinal tissues.^1–3^ When an agonist binds to and activates PXR, PXR forms a heterodimer with the retinoid X receptor and binds to response elements within the promoters and upstream enhancer elements of its transcriptional target genes. Key drug-metabolizing enzymes, such as CYP3A4, and drug efflux transporters, such as MDR1, not only contribute to xenobiotic detoxification but can also decrease the chemosensitivity of cancer cells to certain chemotherapeutic drugs. Thus, upstream signaling mediated by PXR links these drug metabolic enzymes and transporters to disease chemoresistance and progression.

The tumor suppressor p53 is well established as the guardian of the genome, regulating cell cycle arrest, DNA repair, senescence, and apoptosis.^4,5^ p53, a DNA sequence-specific transcription factor, is mainly localized in the nucleus and, once activated, forms a homotetramer that binds DNA at specific response elements within the promoter region of target genes to induce expression of a myriad of genes involved in apoptosis, cell cycle arrest, senescence, DNA repair, and metabolism.^4,6–8^ DNA damage activates p53 to induce either cell cycle arrest to allow for DNA repair or apoptosis if the repair of DNA damage fails.^9–11^ Wild-type p53 regulates the cell cycle by inhibiting cell growth at the G1 phase and is required to sustain G2 arrest after DNA damage.^12–14^ p53 is mutated in approximately 50% of cancers, often preventing apoptosis; tumors with wild-type p53 might also be resistant to apoptosis because of defective apoptotic signaling downstream of p53.

PXR is mainly expressed in liver and intestines. It has also been detected in other normal and malignant human tissues^15–21^ and has an anti-apoptotic role in various cancers.^20,22,23^ PXR protects human colon cancer cells from doxorubicin-induced apoptosis by downregulating the expression of pro-apoptotic genes.^20^ Likewise, activation of PXR promotes the proliferation and migration of colon cancer cells, contributing to the malignant phenotype of these cells.^21^ In addition, nuclear PXR expression correlates with the clinical state of primary human colon cancer, suggesting a clinical correlation between increased PXR expression and reduced cancer survival.^21^ Previously, we reported that p53 interacted with PXR and inhibited its function as a xenobiotic receptor,^24^ but it was unclear how the p53-PXR interaction affected p53 function. Here, we show that PXR carries out its noncanonical anti-apoptotic functions in colon cancer by reducing the binding of p53 to its target gene promoters, thereby inhibiting the tumor-suppressive activity of p53.

## Results

### PXR expression compromises p53 transcriptional activity

**Figure 1:**
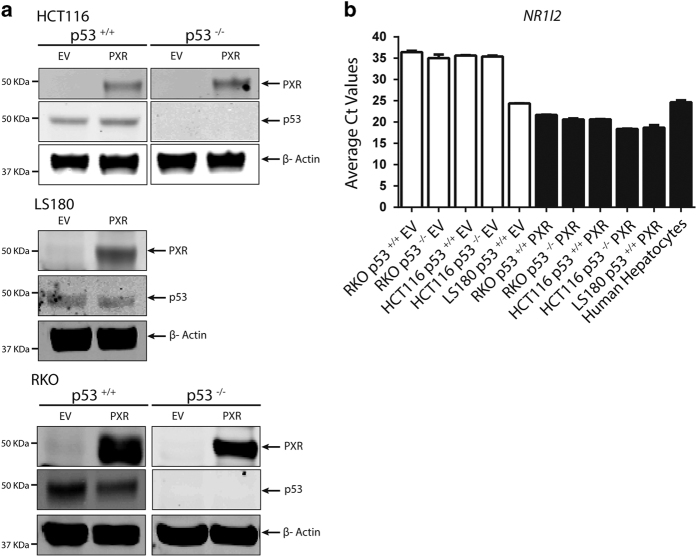
Levels of PXR (endogenous or ectopically expressed) in colon cancer cell models and human hepatocytes. (**a**) Western blot analysis of hPXR (detected by anti-PXR) and p53 (detected by anti-p53) in HCT116 (p53^+/+^, p53^−/−^) cells, LS180 (p53^+/+^) cells, and RKO (p53^+/+^, p53^−/−^) isogenic pairs stably transduced with either lentiviral FLAG-tagged EV or FLAG-tagged hPXR (PXR). *β*-Actin (detected by anti-*β*-actin) was used as a loading control. (**b**) Average C_t_ values from quantitative real-time PCR analysis of hPXR (*NR1I2*) mRNA expression in the RKO isogenic pair (p53^+/+^ (RKO p53^+/+^), p53^−/−^ (RKO p53^−/−^)), the HCT116 isogenic pair (p53^+/+^ (HCT116 p53^+/+^), p53^−/−^ (HCT116 p53^−/−^)), and LS180 (LS180 p53^+/+^) cells stably transduced with EV or PXR and primary human hepatocytes.

The previously reported interaction between PXR and p53^24^, together with the anti-apoptotic^20^ and proliferation-promoting^21^ properties of PXR, prompted us to determine the effects of PXR on p53 function in colon cancer by utilizing human colorectal carcinoma HCT116 and RKO (isogenic pair: p53^+/+^ and p53^−/−^) and human colorectal adenocarcinoma LS180 cell lines, with or without ectopic expression of lentiviral FLAG-tagged empty vector (EV) or FLAG-tagged human PXR (FLAG-hPXR or PXR). The expression of FLAG-hPXR was confirmed via western blot analysis (Figure 1a) and qRT-PCR analysis (Figure 1b). The expression of p53 protein was detected via western blot analysis (Figure 1a). The endogenous PXR level is lower in HCT116 and RKO cells than in LS180 cells and human primary hepatocytes (Figure 1b), consistent with previous studies that correlated higher levels of promoter methylation with lower PXR expression in human colon cancer cells.^25^

**Figure 2:**
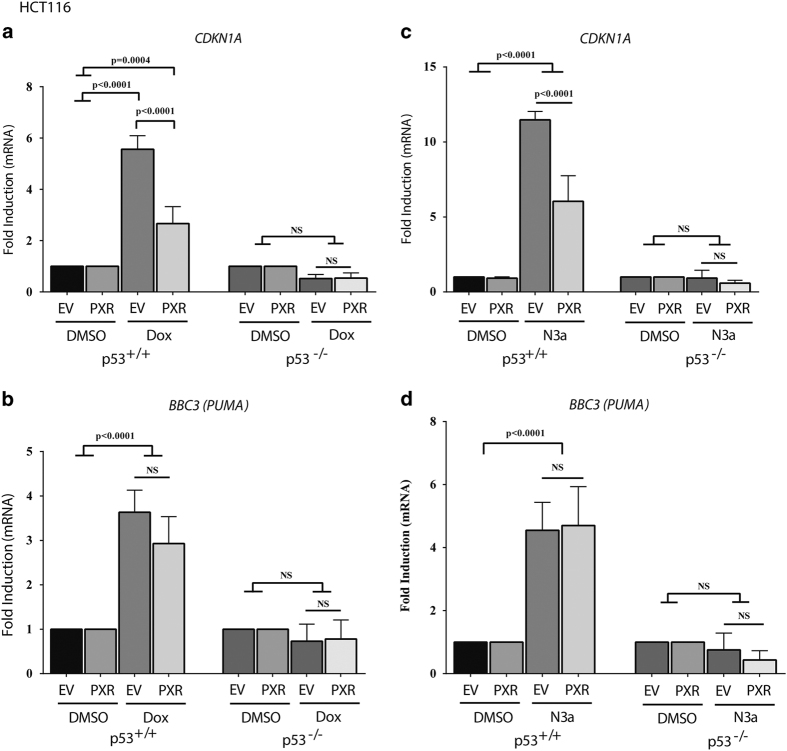
PXR expression reduced mRNA expression of p53 target gene p21 induced by doxorubicin and nutlin-3a. Human colon cancer HCT116 isogenic (p53^+/+^; p53^−/−^) cell lines stably transduced with lentiviral FLAG-tagged EV or FLAG-tagged hPXR (PXR) were treated with DMSO (0.1%) vehicle control, doxorubicin (1 *μ*M) (Dox) for 4 h, or nutlin-3a (10 *μ*M) (N3a) for 24 h. (**a**) qRT-PCR results for p21 (*CDKN1A*) mRNA expression as normalized to *β*-actin in HCT116 isogenic cells treated with DMSO (0.1%) vehicle control or doxorubicin (1 *μ*M). (**b**) qRT-PCR results for PUMA (*BBC3*) mRNA expression as normalized to *β*-actin in HCT116 isogenic cells treated with DMSO (0.1%) vehicle control or doxorubicin (1 *μ*M). (**c**) qRT-PCR results for *CDKN1A* mRNA expression as normalized to *β*-actin in HCT116 isogenic cells treated with DMSO (0.1%) vehicle control or nutlin-3a (10 *μ*M). (**d**) qRT-PCR results for *BBC3* mRNA expression as normalized to *β*-actin in HCT116 isogenic cells treated with DMSO (0.1%) vehicle control or nutlin-3a (10 *μ*M). Data are shown as mRNA fold change (2^-ΔΔCT^) relative to the mRNA level of the corresponding transcript in the control samples as indicated. Experiments were performed at least three times and all samples were analyzed in triplicate. Values are given as means±S.D.s (statistically significant if *P*<0.05, *n*=3). The comparison of experimental conditions was evaluated using one-way ANOVA and Tukey’s multiple comparisons test. The results of a representative experiment are shown.

p21 (*CDKN1A)* is the best-studied transcriptional target of p53.^26–29^ To determine whether FLAG-hPXR expression affected p53 transcriptional activity, we used qRT-PCR to examine the mRNA levels of *CDKN1A* in HCT116 p53^+/+^ cells stably expressing either FLAG-EV (EV) or FLAG-hPXR (PXR) (Figure 2a). After treatment with doxorubicin for 4 h, *CDKN1A* was significantly induced by 5.6- and 2.7-fold in HCTp53^+/+^EV and HCTp53^+/+^PXR cells, respectively, compared with dimethyl sulfoxide (DMSO) treatment (in EV or PXR) (*P*<=0.0004). Exogenous expression of FLAG-hPXR significantly reduced the induction of *CDKN1A* by 52% (*P*<0.0001) (Figure 2a). We also assessed the effects of FLAG-hPXR on the expression of another transcriptional target of p53, PUMA (*BBC3*) (Figure 2b). Doxorubicin significantly induced *BBC3* in HCTp53^+/+^ cells (*P*<0.0001). However, there was no significant difference in the induction of *BBC3* expression by doxorubicin between cells expressing EV and PXR (expression increased by 3.6-fold *versus* 2.9-fold, respectively; *P*=0.1183) (Figure 2b).

Nutlin-3a also significantly induced the expression of *CDKN1A* (Figure 2c) and *BBC3* (Figure 2d) in HCTp53^+/+^ cells (*P*<0.0001). Interestingly, FLAG-hPXR significantly decreased *CDKN1A* expression but not that of *BBC3*. In HCT116 p53^−/−^ cells (Figures 2a–d), both doxorubicin and nutlin-3a failed to induce any p53 target genes, regardless of PXR expression; therefore, we surmise that the effect of PXR on p53 target gene expression is p53 dependent.

To determine whether the effect of PXR on p53 was cell line-specific, we used the RKO isogenic pair (Supplementary Figure 1). The effect of PXR on the expression of *CDKN1A* (Supplementary Figures 1A and 1B) and *BBC3* (Supplementary Figures 1C and 1D) in RKO was similar to that observed in HCT116 cells (Figure 2).

To study the effect of downregulating endogenous PXR, we used LS180 cells, which express a relatively higher level of endogenous PXR and contain wild-type p53 (Figure 1). Exogenous expression of PXR significantly reduced *CDKN1A* expression induced by doxorubicin (Supplementary Figure 2A) but not that induced by nutlin-3a (Supplementary Figure 2B). However, knockdown of PXR in LS180 cells (Supplementary Figure 2C) significantly enhanced both doxorubicin- and nutlin-3a-induced *CDKN1A* expression (Supplementary Figures 2D and 2E). We validated PXR knockdown by using transient transfection with three individual siRNAs (Supplementary Figure 2C), which demonstrated a decrease of approximately 50% in endogenous PXR mRNA expression when compared with that observed with control siRNA (Supplementary Figure 2C). For the drug treatment experiments, we used siRNA PXR ^#^1. Together, these results indicate that elevated levels of PXR compromise the transcriptional activity of p53.

### PXR reduces the occupancy of p53 at its target promoters

**Figure 3:**
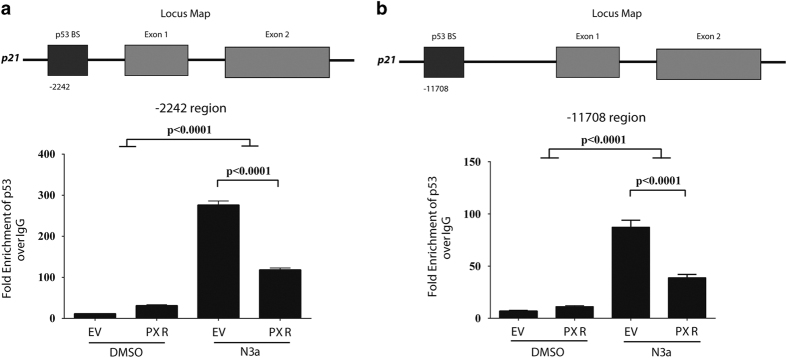
PXR expression reduced p53 recruitment to the p21 promoter mediated by nutlin-3a treatment. HCT116 (p53^+/+^) cells transduced with lentiviral FLAG-tagged EV or FLAG-tagged hPXR (PXR) were treated with DMSO (0.1%) or nutlin-3a (10 *μ*M) for 6 h. (**a**) ChIP analysis of p53 binding to the endogenous −2242 *p21* promoter region in HCT116 (p53^+/+^) cells. (**b**) ChIP analysis of p53 binding to the endogenous distal promoter region at −11 708 on the *p21* gene in HCT116 (p53^+/+^) cells. The fold enrichment was calculated for each treatment sample by normalizing to the respective IgG pull-down (IgG set to 1). The experiments were performed thrice with similar results. Values are given as means±S.Ds. The significance was calculated using one-way ANOVA with Tukey’s correction, and family-wise significance was set to alpha=0.05.

The inhibitory effect of PXR on p53 target gene expression prompted us to determine whether PXR alters p53 occupancy at DNA response elements in the promoter regions of p53 target genes. Accordingly, we performed chromatin immunoprecipitation (ChIP) assays in HCT116 p53^+/+^ cells expressing either EV or exogenous PXR. Table 1 lists the primers used.^30–32^ Two different p53 response elements within the p21 (*CDKN1A*) promoter, as previously characterized, are located −2242 (Figure 3a) and −11 708 bp (Figure 3b) from the p21 transcriptional start site.^30,33^ Nutlin-3a significantly enhanced the occupancy of p53 at both response elements (*P*<0.0001), whereas the occupancy was significantly reduced by exogenous PXR (*P*<0.0001). The inhibitory effect of PXR on p53 occupancy at the *CDKN1A* promoter (Figure 3) is consistent with its effect on *CDKN1A* expression (Figure 2c). PXR also reduced nutlin-3a-induced p53 occupancy at the *MDM2* promoter but not at the *BBC3* promoter (Supplementary Figure 3), consistent with the *MDM2* and *BBC3* gene expression data shown in Supplementary Figure 3 and Figure 2d. These results demonstrate a differential modulatory effect of PXR on the expression of p53 target genes, achieved by reducing the occupancy of p53 at certain target promoters. Supplementary Figure 3D shows the protein levels of p53 and PXR in the lysates used for the ChIP assays.

### PXR expression does not alter CYP3A4 or MDR1 levels

Activating PXR may reduce drug efficacy by inducing the expression of drug-metabolizing enzymes, such as CYP3A4, and drug transporters, such as MDR1 (*ABCB1*).^22,34^ To confirm that PXR was not transcriptionally activated in our cell models (because we did not use a PXR agonist), we examined the expression of *CYP3A4* and *ABCB1* in HCT116 cells expressing either EV or PXR with or without doxorubicin or nutlin-3a treatment. We observed no significant induction of *CYP3A4* in either HCT116 p53^+/+^PXR (*P*>0.9999) or HCT116 p53^−/−^PXR (*P*=0.2659) cells treated with doxorubicin, as compared with respective control cells expressing EV (Supplementary Figure 4A). Similarly, we observed no significant induction of *ABCB1* in HCT116 PXR cells treated with doxorubicin (Supplementary Figure 4B) or nutlin-3a (Supplementary Figure 4D) or of *CYP3A4* in HCT116 PXR cells treated with nutlin-3a (Supplementary Figure 4C). These results confirm that PXR expression reduced p53 activity (induced by doxorubicin or nutlin-3a) by reducing the occupancy of p53 at its target promoters and not by inducing *CYP3A4* or *ABCB1* expression to increase drug metabolism or transport.

### PXR expression promotes malignant transformation and protects cells from doxorubicin and nutlin-3a toxicity

**Figure 4:**
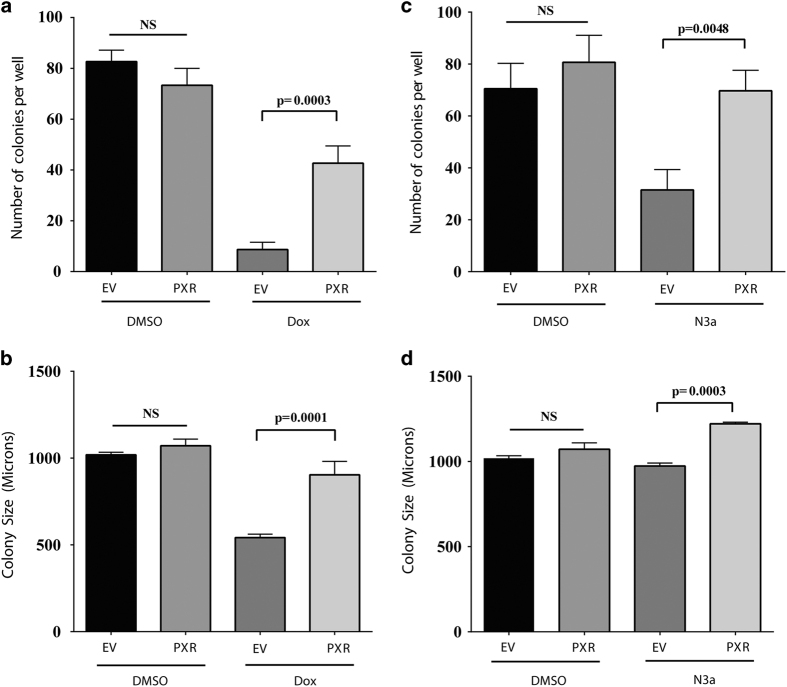
PXR expression protects against doxorubicin and nutlin-3a toxicity contributing to malignant transformation. (**a**) HCT116 (p53^+/+^) cells stably transduced with lentiviral FLAG-tagged EV or FLAG-tagged PXR (PXR) were seeded at 5×10^4^ cells/well and incubated for 10 days with doxorubicin (100 nM) (Dox). The resulting colonies were counted using light microscopy (×4 magnification). (**b**) HCT116 (p53^+/+^) cells stably expressing EV or PXR were incubated for 10 days with doxorubicin (100 nM) (Dox). The size of colony foci (in micrometers) was measured using the ROI perimeter tool in the cellSens software (Olympus). (**c**) HCT116 (p53^+/+^) cells stably transduced with EV or PXR were seeded at 5×10^4^ cells/well and incubated for 10 days with nutlin-3a (1 *μ*M) (N3a). The resulting colonies were counted using light microscopy (×4 magnification). (**d**) HCT116 (p53^+/+^) cells stably expressing EV or PXR were incubated for 10 days with nutlin-3a (1 *μ*M). The size of colony foci (in micrometers) was measured using the ROI perimeter tool in the cellSens software (Olympus). Statistical analysis of treatment groups was carried out using one-way ANOVA followed by Tukey’s multiple comparisons *post hoc* test to find pairwise significance between groups (Prism, GraphPad Software Inc., La Jolla, CA, USA). Values are given as means±S.D.s. Differences were considered statistically significant if the *P*-value was 0.05 or less. The experiments were performed at least three times; the results of a representative experiment are shown.

We further determined the effect of PXR expression on the tumor-suppressive function of p53 by assessing the effects of FLAG-hPXR expression on the malignant transformation of HCT116 p53^+/+^ cells in soft agar (Figure 4). We observed no significant change in the number of colonies formed by HCT116 p53^+/+^EV or HCT116 p53^+/+^PXR cells treated with DMSO (*P*=0.2339) (Figure 4a). Doxorubicin treatment significantly reduced the number of colonies formed in HCT116 p53^+/+^EV and HCT116 p53^+/+^PXR cells by 89.5% and 48.4%, respectively, compared with DMSO-treated HCT116 p53^+/+^EV cells, and by 88.2% and 41.8%, respectively, compared with DMSO-treated HCT116 p53^+/+^PXR cells (*P*=0.0006) (Figure 4a). Importantly, in cells treated with doxorubicin, the expression of FLAG-hPXR (in HCT116 p53^+/+^PXR cells) resulted in a 4.9-fold increase in the number of colonies formed when compared with cells expressing EV (in HCT116 p53^+/+^EV cells) (*P*=0.0003) (Figure 4a).

We also examined the effect of PXR expression on colony size. Although there was no significant difference in colony size between HCT116 p53^+/+^EV and HCT116 p53^+/+^PXR cells treated with DMSO (*P*=0.6153), doxorubicin treatment significantly reduced the colony size of HCT116 p53^+/+^EV cells by 46.8% (*P*<0.0001) but had no significant effect on that of HCT116 p53^+/+^PXR cells (*P*=0.0665), when compared with DMSO-treated HCT116 p53^+/+^EV cells (Figure 4b). The expression of FLAG-hPXR significantly increased the colony size by 66.8% (*P*=0.0001) (comparing HCT116 p53^+/+^PXR to HCT116 p53^+/+^EV cells in the presence of doxorubicin) (Figure 4b). Similarly, the expression of FLAG-hPXR significantly rescued the toxic effect of nutlin-3a, as evidenced by the higher number (Figure 4c, *P*=0.0048) and larger size (Figure 4d,* P*=0.0003) of HCT116 p53^+/+^PXR cell colonies. The effect of PXR expression on rescuing the toxicity of doxorubicin and nutlin-3a was also significant (*P*<0.0001) in RKO p53^+/+^ and LS180 cells (Supplementary Figure 5), as evidenced by the increased number of colonies. The effect on colony size varied depending on the cell model and chemical used.

Taken together, these results demonstrate that PXR contributes to malignant transformation and protects cells from cytotoxicity in the presence of known p53 activators, such as doxorubicin and nutlin-3a.

## Discussion

The evasion of cell death is a hallmark of cancer.^35^ Cancer cells can modulate a response to cellular stress by suppressing DNA repair, altering cell cycle checkpoint control, or, ultimately, downregulating activators of apoptosis, thereby promoting genomic instability and the avoidance of apoptosis.^36^ PXR is well established as a xenobiotic nuclear receptor,^1–3^ but emerging evidence has shown PXR to be a regulator of apoptosis that promotes a malignant phenotype in colon cancer.^20,21^ Because a substantial number of clinical drugs and therapeutic agents act as PXR ligands, the effects of PXR on cellular stress and apoptosis are important and may have biological significance in disease progression. Previous studies demonstrated that PXR protected human colon cancer cells from doxorubicin-induced apoptosis by downregulating pro-apoptotic genes^20^ and that activation of PXR promoted cell proliferation and migration in colon cancer cells, suggesting that PXR promotes the malignant phenotype in colon cancer.^21^

We previously performed a mass spectrometric screen and identified novel protein binders of PXR, including p53, which physically interact with PXR to decrease its activity in inducing drug metabolic enzymes, such as CYP3A4.^24^ However, it was unclear whether the PXR–p53 interaction was mutually inhibitory. As p53 can be activated by DNA damage to induce either cell cycle arrest to allow for DNA repair or, if DNA damage cannot be repaired, apoptosis,^9,10^ the means by which PXR modulates p53-mediated apoptosis following cellular stress warrants further investigation. Given the known roles of PXR in colon tumorigenesis,^20,21^ we hypothesized that the protein–protein interaction between PXR and p53 contributed to the oncogenic functions of PXR and enhanced colon cancer tumorigenicity by inhibiting p53. Our results reveal that the interaction between p53 and PXR is mutually inhibitory in colon cancer cells.

In response to agonist binding, PXR binds to the promoters of its target genes to activate their expression. Our previous studies showed that wild-type p53 binds directly to PXR and decreases PXR recruitment to the *CYP3A4* promoter. In response to cellular stress, such as that induced by doxorubicin, p53 expression is enhanced. Activated p53 binds to its response elements, which follow the consensus sequence motif of 5′-RRRCWWGYYYnRRRCWWGYYY-3′ (where R is purine, Y is pyrimidine, and W is adenine or thymine) to regulate the transcription of its target genes that are involved in apoptosis, cell cycle arrest, and senescence.^8^ In this study, PXR expression decreased p53-binding affinity to response elements within the *p21* (*CDKN1A)* and *MDM2* promoters. Thus, PXR regulates p53 function by modulating p53 recruitment to target gene promoters in response to cellular stress, providing mechanistic insight into the anti-apoptotic roles of PXR in colon cancer.

In our cell models, PXR expression decreases p53 function by reducing p53 occupancy at its target promoters without inducing CYP3A4 or MDR1. These results suggest that PXR inhibits p53 independently of its transactivation activity. Previous studies have shown a ligand-dependent effect of PXR on cell proliferation, promoting the ‘malignant’ phenotype of cancer and reducing apoptosis. Gupta and colleagues^37^ reported that PXR activation promotes cell proliferation and tumor growth mainly through the upregulation of *CYP3A4* and *UGT1A1*. However, these authors also suggest apoptotic gene downregulation as a potential mechanism of resistance in ovarian cancer, which is consistent with our observation of a nongenomic ligand-independent effect of PXR on p53-mediated function and apoptosis. Conversely, Ouyang and colleagues^38^ reported that PXR expression inhibited colon cancer growth in HT29 cells and in female BALB/c mice carrying HT29 xenografts. However, HT29 cells endogenously express mutated p53. In the current study, we utilized HCT116 and RKO isogenic cell line pairs, as well as LS180 cells lines that collectively express functional wild-type p53. Thus, these results suggest that p53 status may have a role in modulating PXR effects on colon cancer growth and interindividual variability in response to chemotherapeutic drugs, particularly those that elicit p53-mediated signaling.

**Figure 5:**
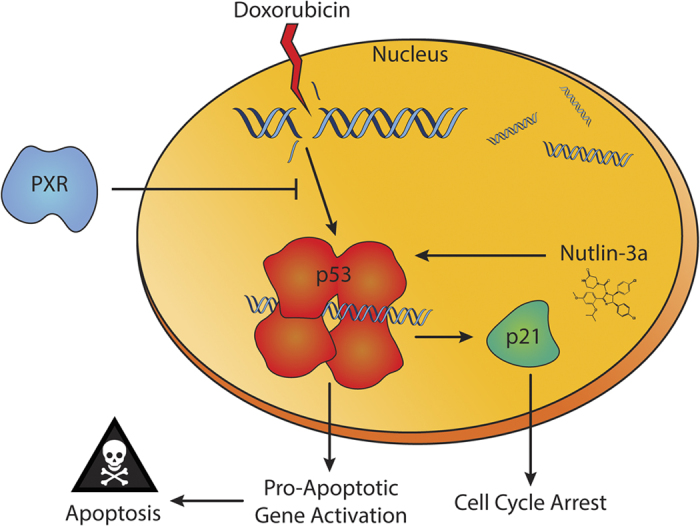
Schematic of PXR–p53 protein–protein interaction. Doxorubicin, a genotoxic chemotherapeutic drug, induces the DNA damage response via p53 activation. Nutlin-3a, a nongenotoxic drug, stabilizes and activates p53 by binding to MDM2 and disrupting the MDM2–p53 interaction. The tumor suppressor p53 can induce transcriptional activation of genes that induce cell cycle arrest, such as p21. In addition, p53 mediates the transcriptional activation of pro-apoptotic genes to induce various mechanisms of apoptosis. Expression of PXR can attenuate the transcriptional function of p53, decreasing the gene expression of cell cycle arrest and pro-apoptotic genes and thereby protecting cells from apoptosis and cell cycle arrest induced by genotoxic and nongenotoxic drugs and promoting malignant transformation.

The regulation of p53 and the expression of its target genes are complex processes involving many different signaling pathways. In our studies, the expression of PXR inhibited the induction of *p21* (*CDKN1A*) and *MDM2* but not of *PUMA* (*BBC3*). The extent to which *CDKN1A* and *MDM2* are inhibited by PXR expression was affected by the specific cellular context and specific activator of p53, further reflecting the complexity of p53 regulation. Figure 5 summarizes the inhibitory effect of PXR on p53 signaling. Nevertheless, our results provide a novel insight into how PXR, in a ligand-independent and nongenomic manner, may be suppressing p53 function in response to chemotherapeutic drugs, rescuing colon cancer cells from drug toxicity, and thus promoting malignant transformation.

## Materials and Methods

### Cell culture, treatments, and viral transduction

The isogenic pair of human colon carcinoma HCT116 (p53^+/+^; p53^−/−^) cells were grown in culture in McCoy’s 5A medium supplemented with 10% fetal bovine serum and 100 units/ml penicillin and 100 *μ*g/ml streptomycin (PenStrep) at 37 °C in 5% CO_2_ as previously described.^24^ Human colorectal adenocarcinoma LS180 cells were obtained from ATCC (Manassas, VA, USA) and grown in culture in Eagle’s minimum essential medium supplemented with 10% fetal bovine serum and PenStrep according to ATCC guidelines. The isogenic pair of human colon carcinoma RKO (p53 ^+/+^; p53^−/−^) cells were obtained from Horizon (Cambridge, UK) and grown in culture in RPMI 1640 medium including 2 mM L-glutamine and 25 mM sodium pyruvate supplemented with 10% fetal bovine serum and PenStrep at 37 °C in 5% CO_2._ RKO (p53^−/−^) cells were maintained under antibiotic selection by using G418 (1 mg/ml). Primary human hepatocytes were obtained through the Liver Tissue Cell Distribution System (Pittsburgh, PA, USA; case numbers 14-008 (M) and 14-005 (F)) or from Triangle Research Laboratories (Research Triangle Park, NC, USA; case number HUM4043 (F)). The primary hepatocytes were maintained in Williams’ Medium E (Sigma–Aldrich, St. Louis, MO, USA) supplemented with primary hepatocyte maintenance supplement (ThermoFisher Scientific, Waltham, MA, USA). HCT116, RKO, and LS180 cells stably expressing hPXR (FLAG-hPXR) were established using the lentiviral CLEG-SF2-GFP/Flag-PXR plasmid (SF2-FLAG-hPXR), as described previously.^39^ For experiments, doxorubicin hydrochloride (44583-1MG, Sigma, St. Louis, MO, USA) was dissolved in DMSO (KP31817, EMD Millipore, Billerica, MA, USA) and diluted from a 1.0 mM stock concentration. Nutlin-3a (SML0580-5MG, Sigma–Aldrich) was dissolved in DMSO (KP31817, EMD Millipore) and diluted from a 10 mM stock concentration.

### siRNA transfection

LS180 cells were seeded (2×10^5^/well) in six-well plates. After seeding, the cells were transfected with either 25 nM control siRNA or 25 nM siRNA targeting PXR (siRNA #1: siGENOME Set of four siRNA D-003415-02, NR1I2, target sequence: GAUGGACGCUCAGAUGAAA; siRNA#2: siGENOME Set of four siRNA D-003415-04, NR1I2, target sequence: CAGGAGCAAUUCGCCAUUA; siRNA#3: siGENOME Set of four D-003415-05, NR1I2, target sequence: GCUCAUAGGUUCUUGUUCC) (Dharmacon, Lafayette, CO, USA) for 24 h by using RNAimax (ThermoFisher Scientific) according to the manufacturer’s instructions. For the qRT-PCR analysis in the drug treatment assay, siRNA #1 was used (siRNA #1: siGENOME Set of four siRNA D-003415-02, NR1I2, target sequence: GAUGGACGCUCAGAUGAAA) (Dharmacon). The transfection efficiency was monitored with the siGLO Red Transfection Indicator (D-001630-02-20; Dharmacon) and was confirmed by qRT-PCR. After 24 h incubation, the medium was replaced with fresh medium, after which the cell cultures were treated with doxorubicin (1 *μ*M), nutlin-3a (10 *μ*M), or DMSO (0.1%) as a vehicle control for 4 h for qRT-PCR experiments.

### Western blot analysis

Cells were harvested by scraping in RIPA lysis buffer (25 mM Tris-HCl, pH 7.6, 150 mM NaCl, 1% NP-40, 1% sodium deoxycholate, 0.1% SDS) with Halt Protease Inhibitor Cocktail (ThermoFisher Scientific), and the lysates were centrifuged at 8000×*g* for 20 min to remove cellular debris, as previously described.^40^ The supernatants were collected as whole-cell lysates. The samples were boiled in sample loading buffer containing SDS, and equal amounts of the samples were loaded onto NuPAGE Bis-Tris 4–12% SDS-PAGE gradient gels (ThermoFisher Scientific), resolved, and transferred to nitrocellulose membranes. The membranes were blocked for 1 h with Odyssey Li-COR Blocking Buffer (LI-COR Biosciences, Lincoln, NE, USA, catalog no. 927-40000) and incubated with the indicated primary antibody overnight at 4 °C. The expression of PXR protein was detected with anti-PXR (H11; mouse monoclonal; sc-48340; Santa Cruz Biotechnology, Dallas, TX, USA). The expression of p53 protein was detected with anti-p53 (B-P3; mouse monoclonal; sc-65334; Santa Cruz Biotechnology). As a loading control, *β*-actin was detected with anti-*β*-actin (Sigma). All western blots were analyzed using the Odyssey Infrared Imaging system (LI-COR Biosciences).

### Quantitative real-time PCR

We used TaqMan assays (ThermoFisher Scientific) to measure mRNA expression, using *β*-actin mRNA as a reference gene. Total RNA extraction from cells was carried out with the Maxwell 16LEV simplyRNA purification kit (Promega, Madison, WI, USA). The mRNA levels were measured by quantitative RT-PCR using the Applied Biosystems 7500HT Fast Real-Time PCR system (ThermoFisher Scientific). The expression of transduced hPXR was validated by real-time qRT-PCR analysis using the PXR-specific TaqMan probe (Hs111426_m1 NR1I2). The expression of p21 (Hs00355782_m1 CDKN1A), MDM2 (Hs01066930_m1 MDM2), PUMA (Hs00248075_m1 BBC3), MDR1 (Hs00184500_m1 ABCB1), and CYP3A4 (Hs00604506_m1 CYP3A4) was validated by qRT-PCR analysis. As an endogenous control, *β*-actin expression was validated by real-time qRT-PCR analysis using the *β*-actin-specific TaqMan probe (Hs01060665_g1 ACTB). The cycle threshold (C_T_) values of each gene of interest, along with that of *β*-actin, were calculated for each sample. The average quantities of the gene transcript were used for calculation purposes. The normalized value was derived by subtracting the C_T_ value of *β*-actin from that of the gene of interest. Data are shown as mRNA fold change (2^-ΔΔCT^) relative to the mRNA level of the corresponding transcript in the control samples as indicated. The experiments were performed at least three times, and all samples were analyzed in triplicate.

### Soft agar colony-formation assay

Five thousand viable cells in 1.0 ml of McCoy’s 5A cell culture medium containing 5% fetal bovine serum, 0.5% PenStrep antibiotics, and 0.3% soft agar were layered on top of a bottom layer consisting of solidified 0.7% agar in McCoy’s 5A medium in six-well plates. The cells were incubated in soft agar for 10 days at 37 °C in 5% CO_2_. Colony foci were counted manually by light microscopy (×4 magnification). The colony foci size (in micrometers) was measured using the ROI perimeter tool in the cellSens software (Olympus, Center Valley, PA, USA). The experiments were performed at least three times in triplicate.

### ChIP assay

HCT116 cells transduced with lentiviral FLAG-tagged EV or FLAG-hPXR (hPXR) were grown in flasks and treated with DMSO (0.1%) or nutlin-3a (10 *μ*M) for 6 h. The proteins were then crosslinked with 1% formaldehyde for 10 min. The cell extracts were digested for 10 min with 50 units of micrococcal nuclease (New England Biolabs, Ipswich, MA, USA) at 37 °C, and then further sonicated to yield sheared DNA fragments with an average length of 200 to 1000 base pairs. The sonicated samples were pelleted by centrifugation, and the supernatant was diluted sixfold with ChIP dilution buffer (0.01% SDS, 1.1% Triton X-100, 1.2 mM EDTA, 16.7 mM Tris-HCl, pH 8.1, 167 mM NaCl, and protease inhibitor cocktail). The samples were precleared with protein G Sepharose 4 Fast Flow (GE Healthcare, Pittsburgh, PA, USA) in the ChIP dilution buffer (1 : 1) and preblocked with 200 *μ*g/ml sheared herring sperm DNA and 500 *μ*g/ml BSA. One hundred microliters of diluted supernatant were reserved as input (10%) for each treatment. The remaining chromatin was then divided and 1 ml each was incubated overnight at 4 °C with either anti-p53 (DO-1, Santa Cruz Biotechnology), anti-RNA polymerase II (clone CTD4H8, EMD Millipore), or control mouse IgG. The antibody-protein-DNA complex was precipitated by incubation with Protein G Sepharose beads for 2 h at 4 °C. The protein-DNA complex was eluted from the beads with elution buffer (1% SDS, 0.1 M NaHCO_3_). The crosslinks were reversed, and DNA was eluted from the protein-DNA complexes by adding 200 mM NaCl and incubating at least overnight at 65 °C. The DNA was recovered and purified after protein digestion with Proteinase K at 45 °C for 2 h. Quantitative RT-PCR was performed to determine the change in p53 and RNA polymerase II occupancy at various known sites of p53 binding. The double-negative controls were nonspecific antibody (normal mouse IgG) and primers coding for regions that do not interact with p53. Thermal cycling conditions were 95 °C for 10 min followed by 45 cycles of 25 s at 95 °C, 30 s at 60 °C, and 30 s at 72 °C. The primer sequences used are listed in Table 1.

### Statistical analysis

The statistical analysis of the treatment groups was carried out using one-way ANOVA followed by Tukey’s multiple comparisons *post hoc* test to find pairwise significance between groups (Prism, GraphPad Software Inc., La Jolla, CA, USA). Values are given as means±S.D. *P*-values of 0.05 or less were considered statistically significant.

## Figures and Tables

**Table 1:** List of primers used in the ChIP assays

*Promoter region amplified*	*Forward primer*	*Reverse primer*	*Reference*
p21, −2242 region	5′- CTG TGG CTC TGA TTG GCT TT-3′	5′- CCC TTC CTC ACC TGA AAA CA-3′	^30^
p21, −11 708 region	5′- GAG TGG GTG GCT CAC TCT TC-3′	5′- CTC GCA TCA GCA ACT CTG G-3′	^30^
PUMA +1313 promoter	5′- TCAGTGTGTGTGTCCGACTGTC-3′	5′- GGCAGGGCCTAGCCCA-3′	^31^
MDM2 promoter	5′- GATTGGGCCGGTTCAGTGG-3′	5′- CACAGCTGGGAAAATGCATGG-3′	^32^
