# Supplementary material for: Human pregnane X receptor compromises the function of p53 and promotes malignant transformation
Source: Cell Death Discov. 2016 Apr 18;2:16023–. doi: 10.1038/cddiscovery.2016.23 (PMC4979430; doi:10.1038/cddiscovery.2016.23)
Supplement: Supplementary Information 1 [file cddiscovery201623-s1.doc]

**Human pregnane X receptor compromises the function of p53 and promotes malignant transformation**

Delira Robbins, Milu Cherian, Jing Wu, Taosheng Chen*

Department of Chemical Biology and Therapeutics, St. Jude Children’s Research Hospital

Memphis, Tennessee

Running title: PXR inhibits p53 function

*Corresponding Author

Taosheng Chen, Ph.D.

Department of Chemical Biology and Therapeutics

St. Jude Children’s Research Hospital

262 Danny Thomas Place

Memphis, TN 38105

Phone: (901) 595-5937; Fax: (901) 595-5715

Email: [Taosheng.chen@stjude.org](mailto:Taosheng.chen@stjude.org)

**A.**

**C.**

**B.**

**D.**

**Supplemental Figure 1. PXR expression compromises p53 transcriptional activity.** Human colon cancer RKO isogenic cells were treated with DMSO (0.1%), doxorubicin (Dox; 1 μM) for 4 h, or nutlin-3a (N3a; 10 μM) for 24 h. A. qRT-PCR results for p21 (*CDKN1A*) mRNA expression as normalized to β-actin in RKO isogenic cells treated with DMSO (0.1%) vehicle control or doxorubicin (1 µM). B. qRT-PCR results for *CDKN1A* mRNA expression as normalized to β-actin in RKO isogenic cells treated with DMSO (0.1%) vehicle control or nutlin-3a (10 µM). C. qRT-PCR results for PUMA (*BBC3*) mRNA expression as normalized to β-actin in RKO isogenic cells treated with DMSO (0.1%) vehicle control or doxorubicin (1 µM). D. qRT-PCR results for *BBC3* mRNA expression as normalized to β-actin in RKO isogenic cells treated with DMSO (0.1%) vehicle control or nutlin-3a (10 µM). Data are shown as mRNA fold change (2^-ΔΔCT^) relative to the mRNA level of the corresponding transcript in the control samples. Experiments were performed at least 3 times, and all samples were analyzed in triplicate. Values are given as means ± SDs (statistically significant if *P* < 0.05, *n* = 3). The comparison of experimental conditions was evaluated by one-way ANOVA and Tukey’s multiple comparisons test. The results of a representative experiment are shown.

**A.**

**B..**

**D.**

**E.**

**C.**

**Supplementary Figure 2. PXR expression compromises p53 transcriptional activity.**A and B. Human colon cancer LS180 cells stably expressing FLAG-tagged empty vector (EV) or FLAG-tagged hPXR (PXR) were treated with DMSO (0.1%), doxorubicin (Dox; 1 μM) for 4 h, or nutlin-3a (N3a; 10 μM) for 24 h. A. qRT-PCR results for p21 (*CDKN1A*) mRNA expression, as normalized to β-actin in LS180 cells treated with DMSO (0.1%) vehicle control or doxorubicin (1 µM). B. qRT-PCR results for *CDKN1A* mRNA expression as normalized to β-actin in LS180 cells treated with DMSO (0.1%) vehicle control or nutlin-3a (10 µM). C. qRT-PCR results for PXR (*NR1I2*) mRNA expression after siRNA knockdown (using 3 individual siRNAs targeting PXR, designated siRNA #1, #2, and #3) as normalized to β-actin in LS180 cells. D. qRT-PCR results for p21 (*CDKN1A*) mRNA expression as normalized to β-actin after control siRNA (Ctrl siRNA) or siRNA knockdown of PXR (PXR siRNA #1) in LS180 cells treated with DMSO (0.1%) vehicle control or doxorubicin (1 µM). E. qRT-PCR results for p21 (*CDKN1A*) mRNA expression as normalized to β-actin after control siRNA (Ctrl siRNA) or siRNA knockdown of PXR (PXR siRNA #1) in LS180 cells treated with DMSO (0.1%) vehicle control or nutlin-3a (10 µM). Experiments were performed at least 3 times, and all samples were analyzed in triplicate. The results of a representative experiment are shown. Values are given as means ± SDs (statistically significant if *P* < 0.05, *n* = 3). The comparison of experimental conditions was evaluated by one-way ANOVA and Tukey’s multiple comparisons test.

**A.**

**B.**

**C.**

**D.**

**EV**

**PXR**

**DMSO**

**DMSO**

**N3a**

**N3a**


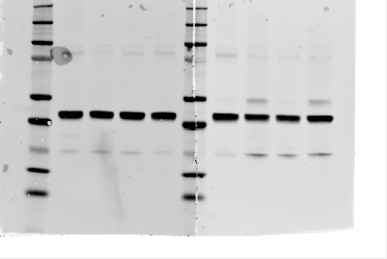


**p53**

**50 kDa**


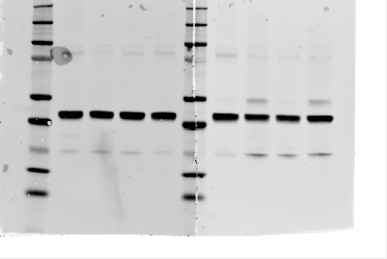


**β-Actin**

**37 kDa**


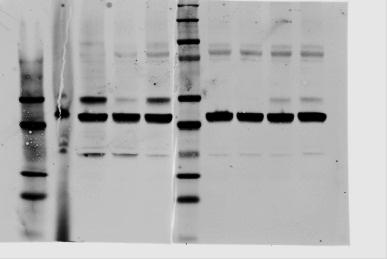


**PXR**

**50 kDa**


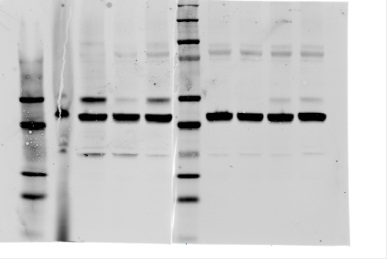


**37 kDa**

**β-Actin**

**Supplemental Figure 3. PXR reduces the occupancy of p53 at its target promoters.** HCT116 (p53^+/+^) cells transduced with lentiviral FLAG empty vector (EV) or FLAG-hPXR (PXR) were grown in flasks and treated with DMSO (0.1%) or nutlin-3a (N3a; 10 μM) for 6 h. A. ChIP analysis of p53 binding on the endogenous *MDM2* promoter region in HCT116 (p53^+/+^) cells. B. ChIP analysis of p53 binding on the endogenous *PUMA* promoter region in HCT116 (p53^+/+^) cells. The fold enrichment was calculated for each of the treatment samples by normalizing to the respective IgG pull-down (IgG set to 1). The significance was calculated by one-way ANOVA with Tukey’s correction, and family-wise significance was set to alpha = 0.05. C. qRT-PCR results for *MDM2* mRNA expression as normalized to β-actin in HCT116 (isogenic pair: p53^+/+^ and p53^−/−^) cells treated with DMSO (0.1%) vehicle control or nutlin-3a (10 µM). D. Protein levels of p53 and PXR (detected using anti-p53 and anti-PXR, respectively) were examined in lysates used for the ChIP assays. Values are given as means ± SDs (statistically significant if *P* < 0.05, *n* = 3). The comparison of experimental conditions was evaluated by one-way ANOVA and Tukey’s multiple comparisons test.

**A.**

**B.**

**C.**

**D.**

**Supplemental Figure 4. PXR expression does not alter CYP3A4 or MDR1 levels.**  Human colon cancer HCT116 isogenic cells stably expressing FLAG-tagged empty vector (EV) or FLAG-tagged hPXR (PXR) were treated with DMSO (0.1%), doxorubicin (Dox; 1 μM) or nutlin-3a (N3a; 10 μM) for 24 h. The cells were then harvested and lysed to assess *CYP3A4* and *MDR1* mRNA expression by qRT-PCR. A. qRT-PCR results for *CYP3A4* mRNA expression as normalized to β-actin in HCT116 isogenic cells treated with DMSO (0.1%) vehicle control or doxorubicin (1 µM). B. qRT-PCR results of *MDR1* mRNA expression as normalized to β-actin in HCT116 cells treated with DMSO (0.1%) vehicle control or doxorubicin (1 µM). C. qRT-PCR results for *CYP3A4* mRNA expression as normalized to β-actin in HCT116 isogenic cells treated with DMSO (0.1%) vehicle control or nutlin-3a (10 µM). D. qRT-PCR results of *MDR1* mRNA expression as normalized to β-actin in HCT116 cells treated with DMSO (0.1%) vehicle control or nutlin-3a (10 µM). Values are given as means ± SDs (statistically significant if *P* < 0.05, *n* = 3). The comparison of experimental conditions was evaluated by one-way ANOVA and Tukey’s multiple comparisons test.

**RKO p53 ^+/+^**

**LS180**

**A.**

**C.**

**E.**

**G.**

**B.**

**D.**

**F.**

**H.**

**Supplemental Figure 5. PXR expression promotes malignant transformation and protects cells from doxorubicin and nutlin-3a toxicity.** A. Colony count for RKO (p53^+/+^) cells stably transduced with FLAG-tagged empty vector (EV) or FLAG-tagged hPXR (PXR), seeded at 5 × 10^4^ cells/well, and incubated with doxorubicin (100 nM) (Dox) for 10 days. The colonies were counted using light microscopy (4× magnification). B. Colony size for RKO (p53 ^+/+^) cells stably expressing EV or PXR colonies, measured after 10 days of incubation with doxorubicin (100 nM) (Dox). C. Colony count for RKO (p53^+/+^) cells stably transduced with empty vector (EV) or human wild-type PXR (PXR), seeded at 5 × 10^4^ cells/well, and incubated for 10 days with nutlin-3a (N3a; 1 µM). The colonies were counted using light microscopy (4× magnification). D. Colony size for RKO (p53^+/+^) cells stably expressing EV or PXR colonies, measured after 10 days of incubation with nutlin-3a (1 µM). E. Colony count for LS180 (p53^+/+^) cells stably transduced with EV or PXR, seeded at 5 × 10^4^ cells/well, and incubated with doxorubicin (100 nM) (Dox). The colonies were counted using light microscopy (4× magnification). F. Colony size for LS180 cells stably expressing EV or PXR colonies, measured after 10 days of incubation with doxorubicin (100 nM) (Dox). G. Colony count for LS180 cells stably transduced with EV or PXR, seeded at 5 × 10^4^ cells/well, and incubated for 10 days with nutlin-3a (1 µM). The colonies were counted using light microscopy (4× magnification). H. Colony size for LS180 cells stably expressing EV or PXR colonies, measured after 10 days of incubation with nutlin-3a (1 µM). The colony foci size was measured (in micrometers) with the ROI perimeter tool in the cellSens software (Olympus). Values are given as means ± SDs (statistically significant if *P* < 0.05, *n* = 3). The comparison of experimental conditions was evaluated by one-way ANOVA and Tukey’s multiple comparisons test.
